# A Case of Premature Parturition, by Puncturing the Membranes in a Deformed Pelvis

**Published:** 1814-08

**Authors:** John Marshall

**Affiliations:** Surgeon


*For the Medical and Physical Journal.*

### *A Case of Premature Parturition, by puncturing the Membranes in a deformed Pelvis;* by JOHN MARSHALL, Esq. Surgeon.

BEFORE entering upon a description of the successful termination of this case, I think it expedient to give the heads of two dreadful labours that preceded, the more satisfactorily to prove the relief obtained by adopting this mode of practice, and shew how much the tediousness of the labour, and the severity of my patient's sufferings, were effectually diminished.

Mrs. H. H. D—— was taken in labour of her first child on Tuesday morning, seven o'clock, on the 5th day of May, 1809, and was not delivered until the Friday morning following, about two o'clock; the child was still-born, and at the commencement of the labour was felt to move distinctly, and with considerable vigour.

On the 13th of September, 1810, she was again taken in labour of her second child, on Saturday night, at eleven o'clock, and was not delivered until the Friday after, at seven in the morning, of a still-born child, which seemed to have but very recently died. After each of these labours, she was so much weakened, that her voice was lost for ten weeks, and she was incapable of walking nearly four months.

On the 8th of October, 1811, I first visited this lady; and, hearing attentively her description of the former labours, I proposed to examine the pelvis, after explaining my reasons and suspicions of the state of the parts concerned in parturition.

On examination I found, by measuring the pelvis, a considerable contraction of the pubis at the symphisis, so as to allow only the middle finger to fill the curve, the capacity of the pelvis at the brim formed a long triangle, causing a preternatural distance from the symphisis pubis to the sacrum: this malversation of the pelvis immediately satisfied me of the great difficulty a full-grown fœtus must have to pass, and the total impracticability of my patient's ever completing the full period of gestation without a result similar to what she before experienced. I calculated, as near as possible, the period she had then completed, and proposed to puncture the membranes of the womb on or about the 19th of November following, which would bring the time to seven months and rather more than a fortnight, with a view to be nearer the eighth than the seventh month, to give the child a better chance of being reared, and to favour the probability of a head presentation.

On the 19th of November, about twelve at noon, I punctured tured the membranes of the womb, by means of a probe half as long again as the usual size, and stouter in proportion, conducting the point with my left hand, along the middle finger of the right hand; the liquor amnii began immediately to run off, and was caught in considerable quantity in an earthen vessel before I left the room, and, upon the patient's moving, continued to come away in larger or smaller quantities until the beginning of her pains, which commenced the next day, twenty-six hours after the operation of puncturing, i. e. at two o'clock in the afternoon. The pains were very feeble, and occurred about every half hour; a quarter before eight in the evening, the real strong pains of labour began, and continued equally strong and frequent until the birth of the child, which took place at a quarter past eleven that night, making the short space of *three hours and a half.* The head presented, and as soon as it escaped the contracted part of the pubis, it required only two or three pains to be delivered. My patient was fully able to suckle her child, and four days after delivery, to use her own words, "she felt as well as if nothing had happened."

I observed during the progressive state of labour, that a considerable quantity of a mucilaginous fluid was given off from the glands situate in the neck of the womb, which appeared to make up for the deficiency of the liquor amnii, and to facilitate the passage of the fœtus. This circumstance I have noticed in every case wherein I have performed the operation. The child is now living, and remarkably strong and healthy.

*Half Moon Street, July* 2, 1814.

